# MAP4K3/GLK in autoimmune disease, cancer and aging

**DOI:** 10.1186/s12929-019-0570-5

**Published:** 2019-10-22

**Authors:** Huai-Chia Chuang, Tse-Hua Tan

**Affiliations:** 1 Immunology Research Center, National Health Research Institutes, 35 Keyan Road, Zhunan, 35053 Taiwan; 2 Department of Pathology & Immunology, Baylor College of Medicine, Houston, TX 77030 USA

**Keywords:** MAP4K3 (GLK), HPK1, Autoimmune disease, Cancer metastasis, Aging, IL-17A, PKCθ, IQGAP1, Autophagy, Verteporfin

## Abstract

MAP4K3 (also named GLK) is a serine/threonine kinase, which belongs to the mammalian Ste20-like kinase family. At 22 years of age, GLK was initially cloned and identified as an upstream activator of the MAPK JNK under an environmental stress and proinflammatory cytokines. The data derived from GLK-overexpressing or shRNA-knockdown cell lines suggest that GLK may be involved in cell proliferation through mTOR signaling. GLK phosphorylates the transcription factor TFEB and retains TFEB in the cytoplasm, leading to inhibition of cell autophagy. After generating and characterizing GLK-deficient mice, the important in vivo roles of GLK in T-cell activation were revealed. In T cells, GLK directly interacts with and activates PKCθ through phosphorylating PKCθ at Ser-538 residue, leading to activation of IKK/NF-κB. Thus, GLK-deficient mice display impaired T-cell-mediated immune responses and decreased inflammatory phenotypes in autoimmune disease models. Consistently, the percentage of GLK-overexpressing T cells is increased in the peripheral blood from autoimmune disease patients; the GLK-overexpressing T cell population is correlated with disease severity of patients. The pathogenic mechanism of autoimmune disease by GLK overexpression was unraveled by characterizing T-cell-specific GLK transgenic mice and using biochemical analyses. GLK overexpression selectively promotes IL-17A transcription by inducing the AhR-RORγt complex in T cells. In addition, GLK overexpression in cancer tissues is correlated with cancer recurrence of human lung cancer and liver cancer; the predictive power of GLK overexpression for cancer recurrence is higher than that of pathologic stage. GLK directly phosphorylates and activates IQGAP1, resulting in induction of Cdc42-mediated cell migration and cancer metastasis. Furthermore, treatment of GLK inhibitor reduces disease severity of mouse autoimmune disease models and decreases IL-17A production of human autoimmune T cells. Due to the inhibitory function of HPK1/MAP4K1 in T-cell activation and the promoting effects of GLK on tumorigenesis, HPK1 and GLK dual inhibitors could be useful therapeutic drugs for cancer immunotherapy. In addition, GLK deficiency results in extension of lifespan in *Caenorhabditis elegans* and mice. Taken together, targeting MAP4K3 (GLK) may be useful for treating/preventing autoimmune disease, cancer metastasis/recurrence, and aging.

## Background

**Fig. 1:**
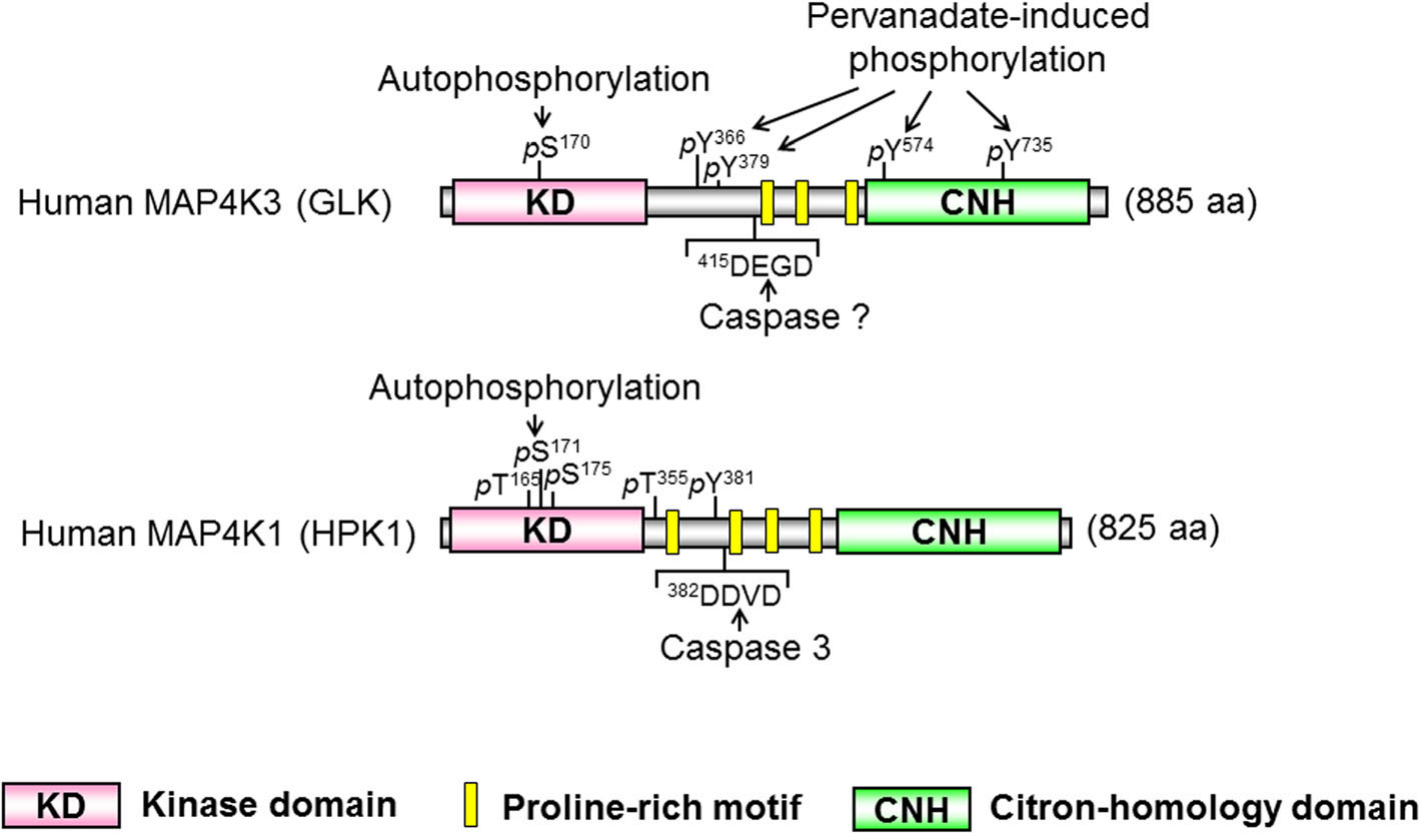
The structural domains of human MAP4K3 (GLK) and human MAP4K1 (HPK1). MAP4K family kinases such as MAP4K3 (GLK) and MAP4K1 (HPK1) are composed of a kinase domain (KD), proline-rich motifs in the middle region, and a citron-homology (CNH) domain. The autophosphorylation sites of GLK and HPK1 are phospho-Ser-170 residue and phospho-Ser-171 residue, respectively. Four pervanadate-induced tyrosine phosphorylation residues on GLK proteins and the known phosphorylation residues on HPK1 proteins are also indicated. The caspase-3 cleavage site on HPK1 and a putative caspase cleavage site on GLK are indicated

**Fig. 2:**
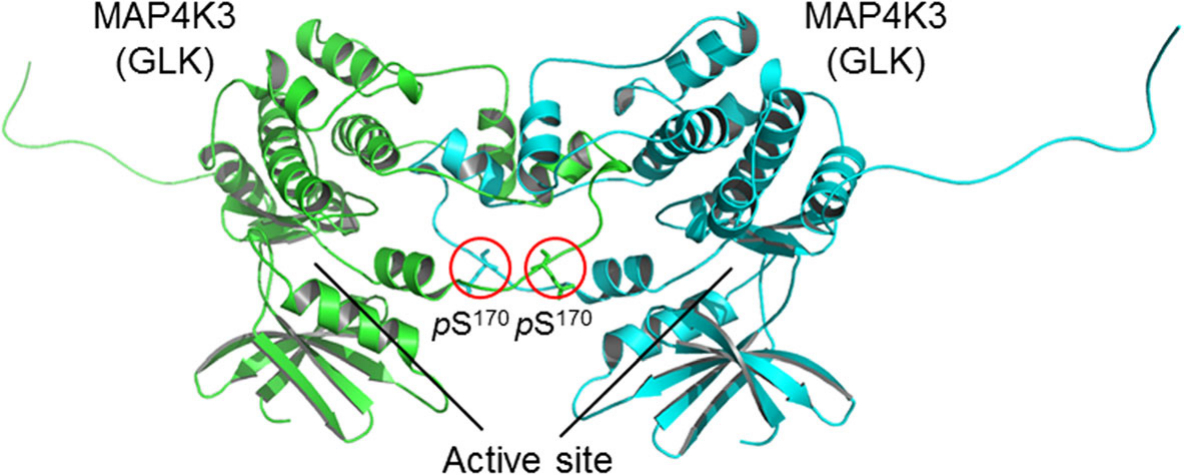
A three-dimensional structure model depicts the dimerization of two MAP4K3 (GLK) kinase domains containing the phospho-Ser-170 residues. Circles denote the phosphates on the Ser-170 residues. The active site of MAP4K3 (GLK) kinase domain is indicated

The MAP4K (MAP kinase kinase kinase kinase or MAPKKKK) family kinases are serine/threonine kinases, which belong to the mammalian Ste20-like kinase family [1, 2]. Overexpression of MAP4K family kinases specifically induces activation of JNK [3–5], but not p38 or ERK [6], in mammalian cells [1]. The MAP4K family contains seven members, including MAP4K1/HPK1 (hematopoietic progenitor kinase 1) [7, 8], MAP4K2/GCK (germinal center kinase)/RAB8IP [9], MAP4K3/GLK (GCK-like kinase) [10], MAP4K4/HGK (HPK1/GCK-like kinase) [11, 12], MAP4K5/KHS (kinase homologous to SPS1/Ste20) [13], MAP4K6/MINK (misshapen/Nck-related kinase) [14], and MAP4K7/TNIK (TRAF2 and NCK interacting kinase) [15]. In 1997, MAP4K3 (GLK) was cloned and identified as a protein kinase that shares 49% amino acid identity with MAP4K1/HPK1 kinase domain and 57% amino acid identity with MAP4K2/GCK kinase domain, thus named GCK-like kinase (GLK) [10]. Like MAP4K1 (HPK1) protein, GLK protein contains a conserved amino-terminal kinase domain, three proline-rich motifs, and a conserved carboxy-terminal citron-homology domain (Fig. 1) [10]. MAP4K1 (HPK1) contains a caspase-3 recognition site (Asp-Asp-Val-Asp, amino acids 382–385) [16]. MAP4K3 (GLK) also contains a consensus sequence (Asp-Glu-Gly-Asp, amino acids 415–418) that matches the substrate sequence Asp-Glu-X-Asp for caspase 2, 3, or 7 [17], suggesting that GLK protein may also be cleaved by a caspase. Overexpression of GLK induces its autophosphorylation and its kinase activity [10]; Ser-170 residue is identified as the trans-autophosphorylation site of GLK protein [18] (Fig. 2). MAP4K3 (GLK) was initially identified as an upstream activator for JNK activation under an environmental stress and proinflammatory cytokines [10]. MAP4K3 (GLK) overexpression induces JNK activation in HEK293T human embryonic kidney cells through MEKK1 [10]. Further studies using knockout/transgenic mice or biochemical approaches reveal additional GLK functions, which are described in this review.

## MAP4K3 (GLK) induces mTOR signaling and inhibits autophagy

**Fig. 3:**
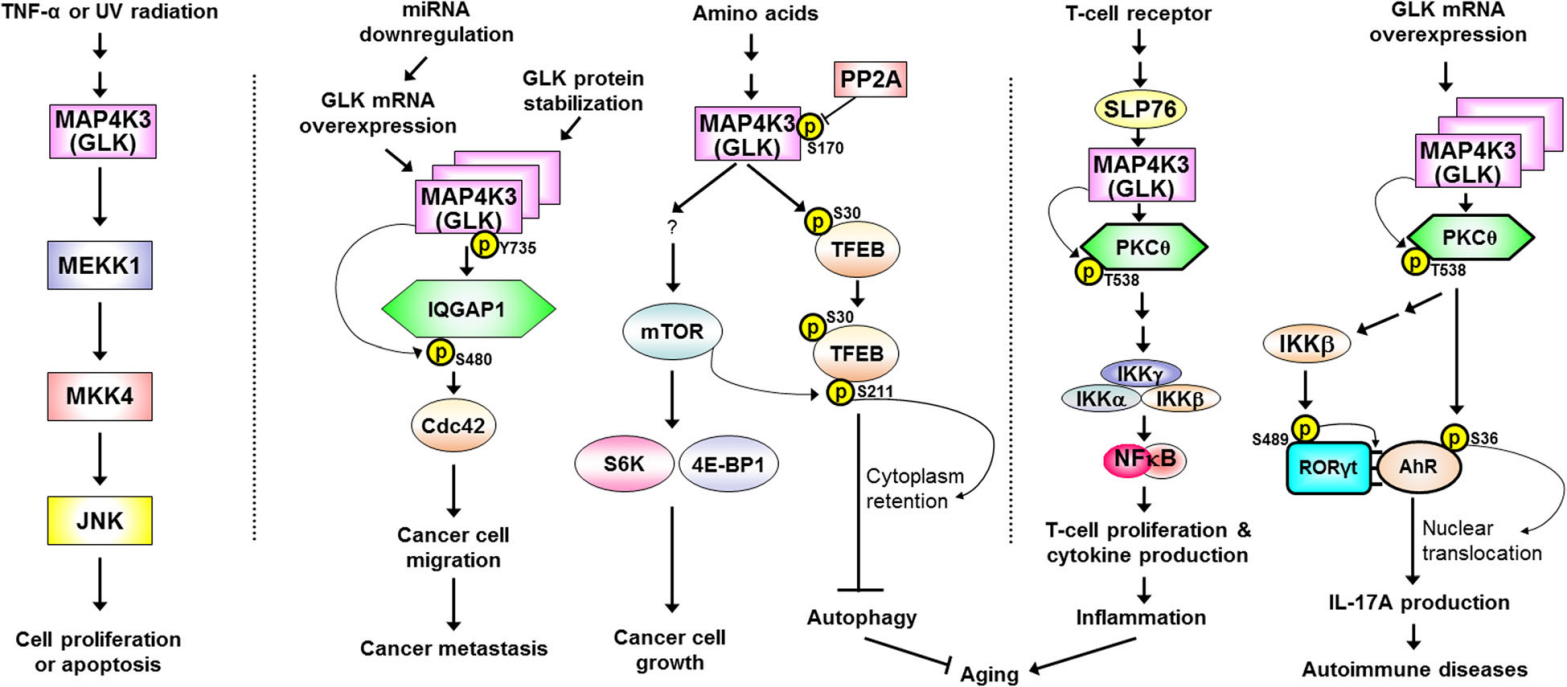
Summary of signal transduction pathways of MAP4K3 (GLK). Arrows denote activation; T bar denotes inhibition. GLK signaling pathways in TNF-α signaling and apoptosis (left panel), cancer and autophagy (middle panel), as well as TCR signaling and autoimmunity (right panel)

MAP4K3 (GLK) controls the cilium growth/development of *Caenorhabditis elegans* and the wing development of *Drosophila melanogaster* through mTOR signaling [19, 20]. Overexpression of MAP4K3 (GLK) induces activation of the mTOR downstream molecules S6K and 4E-BP1 in Hela cells upon sensing cellular nutrient and energy levels; conversely, GLK siRNA knockdown inhibits the activation of S6K and 4E-BP1 [21]. Moreover, like treatment of the mTOR inhibitor rapamycin, GLK siRNA knockdown also inhibits cell growth of Hela cells [21]. In addition, MAP4K3 (GLK) directly interacts with and phosphorylates the transcription factor TFEB at Ser-3 residue, resulting in inhibition of amino acid-depletion-mediated TFEB nuclear translocation [22]. The GLK-induced TFEB Ser-3 phosphorylation is required for the subsequent Ser-211 phosphorylation of TFEB by mTORC1, leading to retention of TFEB in the cytosol and inhibition of cell autophagy [22]. Notably, the GLK-mediated TFEB inactivation facilitates the mTOR-inhibited autophagy pathway, but the TFEB inactivation is not regulated by mTOR signaling [22]. Besides induction of mTOR signaling, GLK overexpression induces NF-κB activation [23] and cell proliferation in primary human hepatocytes [24]. These findings suggest that MAP4K3 (GLK) plays critical roles in promoting cell growth and blocking autophagy (Fig. 3).

## Upstream regulators of MAP4K3 (GLK)

Upon amino acid withdrawal, the phosphatase PP2A directly interacts with and dephosphorylates GLK at the autophosphorylation site Ser-170, leading to inactivation of GLK and GLK-induced mTOR signaling [18]. Besides serine phosphorylation, tyrosine phosphorylation of GLK is induced by EGF stimulation in A549 lung cancer cell lines, suggesting that EGF receptor (EGFR) signaling regulates GLK function or activity [25]. Moreover, Tyr-366, Tyr-379, Tyr-574, and Tyr-735 are identified as the induced tyrosine-phosphorylation residues of GLK in cells treated with the tyrosine phosphatase inhibitor pervanadate [26]. In addition, the adaptor protein HIP-55 (also named mAbp1 and SH3P7) has been identified as an interacting protein of MAP4K3 (GLK) and HPK1 (MAP4K1) [27]. Both GLK and HIP-55 are required for T-cell activation [23, 28]. Furthermore, HIP-55 overexpression stimulates HPK1 kinase activity [27]; conversely, T-cell receptor (TCR)-induced HPK1 activation is reduced in HIP-55 knockout T cells [28]. The data suggest that GLK may also be a downstream molecule of HIP-55. Like HPK1 [29], GLK directly interacts with the adaptor protein SLP-76 under TCR signaling [23]. SLP-76 is required for TCR-induced GLK kinase activity [23]; however, the kinase that phosphorylates and regulates GLK has not been identified.

## MAP4K3 (GLK) controls T-cell activation and T-cell-mediated autoimmune responses

After generating and characterizing GLK-deficient mice, the in vivo roles of GLK in T-cell activation have been revealed. GLK-deficient mice display impaired T-cell-mediated immune responses [23]. In T cells, GLK kinase activity is induced by T-cell receptor (TCR) stimulation [23]. Under TCR signaling, GLK directly interacts with and activates PKCθ through phosphorylating PKCθ at Thr-538 residue but not Ser-676 and Ser-695 residues [23, 30], leading to activation of IKK/NF-κB [23]. In vitro Th1, Th2, or Th17 differentiation is reduced by GLK deficiency [23]. In contrast, suppressive function of GLK-deficient Treg cell is enhanced compared to that of wild-type Treg cell [23]. Thus, GLK positively regulates T-cell activation and T-cell function by activating the PKCθ-IKK pathway (Fig. 3).

GLK overexpression contributes to autoimmune responses. GLK-deficient mice display decreased disease scores in autoimmune disease models such as collagen-induced arthritis (CIA) [31] and experimental autoimmune encephalomyelitis (EAE) [23]. Consistently, the percentage of GLK-overexpressing T cells is enhanced in the peripheral blood from patients with human autoimmune diseases, including systemic lupus erythematosus (SLE) [23], rheumatoid arthritis (RA) [31], and adult-onset Still’s disease (AOSD) [32]. The GLK-overexpressing T cell population is correlated with disease severity of autoimmune disease patients [23, 31, 32]; therefore, GLK-overexpressing T cell is a biomarker for autoimmune diseases.

To mimic GLK overexpression in human autoimmune patient T cells, T-cell-specific GLK transgenic mice have been generated and characterized. The transgenic mice spontaneously develop autoimmune diseases and systemic inflammation [33]. The pathogenic cytokine IL-17A levels are specifically increased in the sera of T-cell-specific GLK transgenic mice [33]. Consistently, GLK overexpression co-exists with IL-17A overproduction in peripheral blood T cells from human SLE patients [34]; the GLK^+^IL-17A^+^ T cell population is a diagnostic biomarker for active SLE [34]. The pathogenic mechanism of autoimmune disease by GLK overexpression has been unraveled using several gene knockout/transgenic mice and biochemical approaches [33]. In T cells, GLK overexpression induces activation of PKCθ and IKKβ [33]. IKKβ phosphorylates RORγt at Ser-489 residue, leading to the interaction between the transcription factors RORγt and AhR [33]. On the other hand, PKCθ phosphorylates AhR at Ser-36 residue, resulting in nuclear translocation of the AhR-RORγt complex [33]. Thus, GLK overexpression in T cells selectively promotes IL-17A transcription by inducing the AhR-RORγt complex (Fig. 3). The GLK-regulated AhR-RORγt complex is also induced in peripheral blood T cells from human SLE patients [34]. Conversely, AhR or RORγt deficiency results in attenuation of autoimmune responses of T-cell-specific GLK transgenic mice [33]. These findings indicate that GLK signaling and GLK-induced AhR-RORγt complex are potential therapeutic targets for SLE.

## MAP4K3 (GLK) promotes cancer metastasis/recurrence

GLK overexpression occurs in cancer tissues of human non-small cell lung carcinoma (NSCLC) [35], hepatocellular carcinoma (HCC) [24], glioblastoma [36], and papillary thyroid carcinoma (PTC) [37]. One publication reported that GLK immunohistochemistry (IHC) staining signals per square microns are decreased in cancer tissues of pancreatic cancer patients [38]. Nevertheless, IHC staining intensity of GLK seems to be more condensed in ductal adenocarcinoma-like cells, which would be consistent with others’ findings that GLK is overexpressed in several cancer tissues [24, 35–37]. Moreover, a GLK somatic mutation, encoding E351K, has been identified in human pancreatic cancer [39]. The GLK E351K mutant displays higher kinase activity than that of wild-type GLK [26], indicating that GLK is an oncogene involved in tumorigenesis of human pancreatic cancer.

For human NSCLC and HCC, GLK overexpression in cancer tissues is correlated with cancer recurrence [24, 35]. The predictive power of GLK overexpression for cancer recurrence is higher than that of pathologic stage. Another MAP4K member, MAP4K4/HGK, induces cell migration and promotes cancer metastasis [40]. It is likely that GLK may also promote cancer metastasis by enhancing cell migration. In addition, overexpression of the microRNAs let-7c, miR-199-a-5p, or miR-206 inhibits GLK expression in cell lines by targeting the GLK 3’UTR [37, 41, 42]. Overexpression of let-7c or miR199-a-5p also inhibits cell migration and invasion of cancer cell lines [41, 42]. Consistently, cell migration and invasion are also attenuated by GLK siRNA knockdown but enhanced by GLK overexpression in liver cell lines [42]. Moreover, the two aforementioned miRNAs (let-7c and miR-199-a-5p) are downregulated in tumor tissues of human NSCLC and HCC, respectively, and the downregulation of these two miRNAs is correlated with poor outcome of cancer patients [41, 42]. These results suggest that GLK may induce cancer metastasis/recurrence of NSCLC and HCC by enhancing cell migration/invasion.

A recent publication reveals the mechanism of GLK-induced cell migration and cancer metastasis using whole-body GLK transgenic mice [26] (Fig. 3). GLK transgene induces cell migration in primary lung epithelial cells [26]. GLK transgene does not initiate tumorigenesis in mice [26]; however, GLK transgene promotes distant cancer metastasis in a genetically modified lung cancer mouse model—lung-specific EGFR-deletion mutant transgenic mouse line (EGFR^del^ Tg) [26]. The GLK-interacting protein IQGAP1 is responsible for GLK-induced cell migration and cancer metastasis [26]. GLK directly interacts with and phosphorylates IQGAP1 at Ser-480 residue, resulting IQGAP1 activation and subsequent cell migration [26]. Furthermore, Ser-480 phosphorylated IQGAP1 induces activation of Cdc42, which controls non-directional/random cell migration [26]. In contrast, GLK-phosphorylated IQGAP1 does not activate another IQGAP1-downstream molecule, Rac1, which controls directional/persistent cell migration. In addition, the direct interaction between GLK and IQGAP1 is mediated by two proline-rich regions of GLK and the WW domain of IQGAP1; this interaction also is inducible by GLK Tyr-735 phosphorylation [26]. GLK and IQGAP1 colocalize at the leading edge of migrating cells [26]. Consistently, GLK-IQGAP1 interaction and GLK-induced IQGAP1 Ser-480 phosphorylation are detectable in cancer tissues and metastatic cancer cells of human lung cancer patients; this interaction and IQGAP1 phosphorylation are correlated with poor survival of lung cancer patients [26]. Conversely, the distant cancer metastasis is abolished by IQGAP1 knockout in a cancer mouse model [26]. Collectively, the GLK-IQGAP1 complex and IQGAP1 Ser-480 phosphorylation are prognostic biomarkers and potential therapeutic targets for human lung cancer recurrence.

Besides intrinsic pathways, GLK overexpression may contribute to metastasis through proinflammatory cytokines. GLK overexpression in T cells induces production of IL-17A [33], which promotes cancer cell migration and increases cancer metastasis [43–45]. Therefore, GLK overexpression in T cells or other cell types may also induce overproduction of IL-17A in tumor microenvironment, leading to cancer metastasis.

## GLK inhibitors for treatment of Th17-mediated autoimmune diseases

GLK overexpression is a therapeutic target for autoimmune diseases and cancer recurrence. Inhibition of GLK may be useful for treating cancer and autoimmune disease patients. An analogue of crizotinib (compound #1) has been identified as a small-molecule GLK inhibitor that binds to the active site of the GLK kinase domain [46]. The IC50 for GLK kinase activity by the crizotinib analogue is 10 nM; however, the IC50 for MAP4K4 (HGK) is 0.8 nM [46]. MAP4K4 (HGK) is a negative regulator for Th17 development [47–49]; therefore, preferential inhibition of MAP4K4 (HGK) by this crizotinib analogue (compound #1) may result in the adverse effect of inducing Th17-mediated immune responses, such as autoimmunity. These results suggest that this crizotinib analogue may not be useful for treatment of autoimmune disease. Another crizotinib analogue (compound #44) was also identified as a GLK inhibitor with IC50 of 3 nM, but it still inhibits other MAP4K members [50]. Furthermore, in vivo clearance rate in animals of this crizotinib analogue (compound #44) is too high to test its inhibitory effects using animal models [50]. Thus, the authors concluded that no promising GLK inhibitors have been developed from the analogues of crizotinib [50].

Recently, an FDA-approved drug, verteporfin, has been identified as a new small-molecule GLK inhibitor [34]. Verteporfin is a light-activated drug for macular degeneration of eyes [51]. The IC50 of verteporfin for GLK kinase activity is 1.15 nM without any photochemical process, while the IC50 of verteporfin for HPK1 (MAP4K1) kinase activity is 7.91 nM [34]. Notably, the IC50 of verteporfin for MAP4K3 (GLK) is the lowest compared to that of other MAP4K members [34]. The verteporfin treatment reduces disease severity in three autoimmune mouse models, including EAE, CIA, and T-cell-specific GLK transgenic mice [34]. Moreover, the verteporfin treatment also efficiently inhibits GLK-induced AhR-RORγt complex and IL-17A production in human SLE T cells [34]. Thus, verteporfin may be repositioned as a novel small-molecule therapeutic drug for Th17-mediated autoimmune diseases.

## GLK inhibitors for treatment of cancer recurrence

The correlation between GLK overexpression and cancer recurrence and the promotion of cancer metastasis by GLK suggest that the GLK inhibitor verteporfin is also useful for treating cancer. Furthermore, verteporfin has also been used in clinical trials for pancreatic cancer due to its inhibitory effect on angiogenesis by releasing reactive oxygen radicals [52]. Thus, verteporfin is a potential therapeutic drug for both autoimmune disease and cancer recurrence.

Natural products also contains GLK inhibitors [53]. Astragalus polysaccharide (APS) and 10-hydroxycamptothecin (HCPT) have been reported as GLK inhibitors that suppress GLK kinase activity and GLK-induced mTOR signaling [53]. Combination treatment of APS and HCPT induces cell apoptosis and reduces cell migration/invasion in H1299 lung cancer cells [53]. Further purification of APS and HCPT may help development of small-molecule GLK inhibitors for treatment of cancer recurrence and autoimmune diseases.

## GLK and HPK1 dual inhibitors for cancer immunotherapy

A higher dose (7.91 nM) of the small-molecule GLK inhibitor verteporfin also inhibits MAP4K1 (HPK1) [34], indicating that verteporfin is a GLK and HPK1 dual inhibitor. Because HPK1 is a negative regulator of T-cell receptor signaling [29, 54] and B-cell receptor signaling [55], HPK1 inhibitors may be used as immune-boosting agents for anti-tumor immunity or vaccination [1]. Thus, combination treatments of HPK1 inhibitors with anti-PD-1 antibody may be effective for cancer combination immunotherapy. Notably, cancer immunotherapy usually induces autoimmune responses, which would be potentially reduced by a GLK inhibitor. Thus, verteporfin is also likely to be a potential therapeutics for cancer immunotherapy due to its suppressive effects on both cancer progression and autoimmune diseases. Furthermore, screening GLK inhibitors would help identification of GLK and HPK1 dual inhibitors for cancer immunotherapy in the future.

## GLK deficiency results in prevention of aging

Besides cell growth, cell proliferation, and cell migration, GLK also regulates animal lifespan. GLK deficiency in *Caenorhabditis elegans* results in an expansion of the worm lifespan [56]. Similarly, GLK-deficient mice show a significant extension of lifespan [34]. The phenotypes of GLK-deficient mice are normal and healthy. The serum levels of proinflammatory cytokines are increased in aged wild-type mice, but are decreased in aged GLK-deficient mice [34]. Chronic inflammation plays a critical role in the aging process. Thus, expanded lifespan of GLK-deficient mice may be due to decreased inflammatory responses (inflamm-aging), suggesting that GLK inhibitor may have anti-inflamm-aging effect. Furthermore, these findings suggest that treatments of human patients using GLK inhibitors may have additional beneficial effects. Nevertheless, we could not rule out the possibility that GLK inhibitors may have potential side effects of attenuated immunity against microbial infections.

## Discussions and conclusions

GLK overexpression in T cell is a critical pathogenic factor for development of autoimmune diseases. Deficiency of GLK or GLK-downstream molecules (such as PKCθ, AhR, RORγt) inhibits disease severity in autoimmune disease mouse models, indicating that GLK signaling is a therapeutic target for autoimmune disease. GLK-induced AhR/phospho-RORγt complex selectively stimulates IL-17A gene transcription; therefore, inhibition of GLK signaling or AhR/phospho-RORγt complex (such as verteporfin treatment) abolishes IL-17A production but maintains physiological functions of other cytokines. Moreover, GLK^+^ Th17 cell can be used as a biomarker to help selection of the SLE patient subpopulation (GLK^high^IL-17A^high^) that is responsive to IL-17A blockade or GLK inhibitors, leading to precision medicine for SLE.

GLK induces cell growth of cancer cells [21, 24]; however, whole-body GLK transgenic mice do not spontaneously develop any cancer [26]. These findings suggest that GLK contributes to tumorigenesis after cancer initiation/transformation. Besides enhancement of cell growth, inhibition of cell autophagy by GLK signaling may be also involved in cancer progression [22]. GLK overexpression in cancer tissues is highly correlated with cancer recurrence [24, 35]. In cancer cells, GLK directly phosphorylates and activates IQGAP1, resulting in induction of Cdc42-mediated cell migration and cancer metastasis [26]. Furthermore, cancer cell migration is blocked by inhibition of GLK activity or disruption of the GLK-IQGAP1 complex [26]. Treatment of natural-product GLK inhibitors or overexpression of the miRNAs targeting GLK inhibits cancer cell migration and invasion in cancer cell lines [41, 42, 53]. The GLK-IQGAP1 complex formation and IQGAP1 Ser-480 phosphorylation in cancer cells are correlated with poor survival of human lung cancer patients. These findings suggest that GLK signaling or GLK-induced IQGAP1 phosphorylation is a prognostic biomarker and therapeutic target for cancer metastasis/recurrence.

T cells from autoimmune disease patients display increased GLK mRNA levels, suggesting that transcription factors/repressors, histone-modifying enzymes, DNA methyltransferases, microRNAs, and/or long-non-coding RNAs (lncRNAs) may be responsible for GLK overexpression. The downregulation of three identified GLK miRNAs in cancer tissues [37, 41, 42] supports that GLK overexpression in cancers is due to downregulation of the miRNAs that target GLK 3’UTR. In addition, sixteen 5′ UTR SNPs, fifty 3′UTR SNPs, and eighty missense SNPs of GLK in cancer patients can be found through NCBI (National Center for Biotechnology Information) website. Moreover, about 240 gene variants of GLK are detected in multiple cancers through COSMIC (Catalogue Of Somatic Mutations In Cancer) website (https://cancer.sanger.ac.uk/cosmic/search?q=MAP4K3). One of these gene variants of GLK, GLK E351K [39], results in enhancement of GLK kinase activity in cancer cells [26]. Furthermore, GLK mRNA levels in cancer tissues of human NSCLC patients are comparable to those of normal adjacent tissues [35], suggesting that GLK protein stability is enhanced in lung cancer cells by an unknown regulatory mechanism. Collectively, it will be interesting to study whether other gene variants can result in induction of GLK mRNA levels, protein stability, or kinase activity in human autoimmune disease or cancer patients. Investigation of regulatory mechanisms of GLK overexpression in autoimmune disease T cells or cancer tissues may help identification of additional therapeutic targets for these diseases.

Collectively, overexpression of GLK induces autoimmune disease and cancer metastasis. Conversely, inhibition of GLK signaling attenuates disease progression of both autoimmune disease and cancer metastasis. Thus, GLK inhibitors could be useful therapeutics for autoimmune disease, as well as cancer recurrence without induction of autoimmune responses [57]. Furthermore, GLK deficiency results in extension of lifespan, suggesting that GLK inhibitors may also have anti-aging effects by attenuating inflammatory responses.

## Data Availability

Data and materials related to this work are available upon request.

## Abbreviations

4E-BP1: eIF4E-binding protein 1
AOSD: Adult-onset Still’s disease
APS: Astragalus polysaccharide
Cdc42: Cell division control protein 42
CIA: Collagen-induced arthritis
EAE: Experimental autoimmune encephalomyelitis
GCK: Germinal center kinase
GLK: GCK-like kinase
HCC: Hepatocellular carcinoma
HCPT: 10-hydroxycamptothecin
HGK: HPK1/GCK-like kinase
HPK1: Hematopoietic progenitor kinase 1
IC50: Half maximal inhibitory concentration
IKK: IκB kinase
IQGAP1: IQ motif-containing GTPase-activating protein 1
KHS: Kinase homologous to SPS1/Ste20
MAP4K: MAP kinase kinase kinase kinase
NSCLC: Non-small cell lung carcinoma
PKCθ: Protein kinase C-theta
PTC: Papillary thyroid carcinoma
RA: Rheumatoid arthritis
S6K: S6 kinase
SLE: Systemic lupus erythematosus
SNP: Single nucleotide polymorphism
TFEB: Transcription factor EB
